# Application of Novel Anion-Exchange Blend Membranes (AEBMs) to Vanadium Redox Flow Batteries

**DOI:** 10.3390/membranes8020033

**Published:** 2018-06-19

**Authors:** Hyeongrae Cho, Henning M. Krieg, Jochen A. Kerres

**Affiliations:** 1 Institute of Chemical Process Engineering, University of Stuttgart, 70199 Stuttgart, Germany; Hyeongrae.cho@icvt.uni-stuttgart.de; 2 Faculty of Natural Science, North-West University, Focus Area: Chemical Resource Beneficiation, Potchefstroom 2520, South Africa; henning.krieg@nwu.ac.za

**Keywords:** anion-exchange blend membrane, vanadium redox flow battery, vanadium ions cross-over, charging–discharging test, coulombic efficiency

## Abstract

Both cation-exchange membranes and anion-exchange membranes are used as ion conducting membranes in vanadium redox flow batteries (VRFBs). Anion-exchange membranes (AEMs) are applied in vanadium redox flow batteries due to the high blocking property of vanadium ions via the Donnan exclusion effect. In this study, novel anion-exchange blend membranes (AEBMs) were prepared, characterized, and applied in VRFBs. Bromomethylated poly(2,6-dimethyl-1,4-phenylene oxide), poly[(1-(4,4′-diphenylether)-5-oxybenzimidazole)-benzimidazole] (PBI-OO) and sulfonated polyether sulfone polymer were combined to prepare 3-component AEBMs with 1,2,4,5-tetramethylimidazole (TMIm) for quaternization. 3-component AEBMs showed significantly enhanced chemical and mechanical properties compared with those of 2-component AEBMs, resulting in an improved performance in VRFBs. The compositions of the anion-exchange polymers in 3-component AEBMs were systematically varied to optimize the AEBMs for the redox-flow battery application. While the 3-component AEBMs showed comparable efficiencies with Nafion^®^ 212 membranes, they displayed improved vanadium ions cross-over as was confirmed by open circuit voltage tests and capacity fade tests conducted in VRFBs. In addition, one of the synthesized 3-component AEBM had a superior coulombic efficiency and capacity retention in a charging–discharging test over 300 cycles at a current density of 40 mA/cm^2^. It can thus be concluded that 3-component AEBMs are promising candidates for long-term operation in VRFBs.

## 1. Introduction

Vanadium redox flow batteries (VRFBs) provide a promising technology for large-scale energy storage. Vanadium redox flow batteries can be described as electrochemical devices that store energy in two separated solutions containing different redox couples. In VRFBs, vanadium ions, which are stored separately (V^2+^/V^3+^ and V^4+^/V^5+^) in electrolyte reservoirs, undergo electrochemical redox reactions on the surface of electrodes [1,2]. Since ion conducting membranes are used in redox flow batteries (RFBs) as a separator between the two electrolytes, they are an integral component of VRFBs. Their role however is not only to conduct semi-permeable ions such as H^+^ for cation-conducting membranes or SO_4_^2−^ and HSO_4_^−^ for anion-conducting membranes thereby maintaining charge balance, but also to prevent the cross-mixing of the two electrolytes (V^2+^/V^3+^ and V^4+^/V^5+^).

For such VRFB separators, both cation-exchange membranes (CEMs) and anion-exchange membranes (AEMs) can be used. The most widely studied CEMs are based on perfluorinated polymer membranes known as Nafion^®^ (developed by DuPont), which are commonly used in VRFBs because of their high proton conductivity and excellent chemical stability [3]. However, in VRFBs, Nafion^®^ displays high vanadium ion permeation, resulting in fast capacity reduction in cycling tests [4]. To reduce the vanadium ions permeability, Nafion^®^ has been modified both externally, for example by coating with polyethyleneimine [5], poly(diallyldimethylammonium chloride) (cationic layer), and poly(styrenesulfonic acid) (anionic layer) [6], or internally, for example by manufacturing composite membranes using inorganic particles [7,8]. However, in spite of these advances, Nafion^®^ remains very expensive, limiting the large-scale commercialization of this technology [9]. For this reason, the development of alternative membranes for VRFBs has been intensively studied. For example, poly(vinylidene fluoride) (PVDF) membranes grafted onto polystyrene displayed better performances than Nafion^®^ membranes [10]. In addition, aromatic main-chain polymers have been intensively studied due to their high chemical stability which can be traced back to their aromatic structure. One such polymer, sulfonated poly(ether ether ketone) (S-PEEK), when applied in VRFBs, has shown only a slight efficiency decrease after 80 charging–discharging cycles at 40 mA/cm^2^ [11]. In contrast, sulfonated Radel^®^-based polymer membranes suffered from significant capacity loss after 40 charging–discharging cycles at a current density of 50 mA/cm^2^, probably due to a radical attack of highly-oxidizing vanadium oxoperoxide species on the electrophilic arylene ether bond [12,13]. Therefore, partially fluorinated aryl CEMs have been developed which possess lower electron density due to pendent strong electron-attracting F moieties, which leads to better stability resulting in improved efficiencies of RFBs [14,15].

Another strategy to improve the performance of VRFBs is using acid-base blend membranes composed of a sulfonated aryl polymer and a basic polymer with aryl group-based polymers [16]. These types of blend membranes, developed by the group of Dr. Kerres over more than 15 years of research, displayed improved chemical and mechanical stability compared to membranes consisting of homopolymers [17,18,19]. In terms of VRFBs, preliminary investigations of the performance of acid-base blend membranes showed higher efficiencies than that of Nafion^®^ [16].

Most AEMs possess positively charged functional groups such as quaternized ammonium (fixed ions) with accompanying and exchangeable anions as counter ions. Through this structure, the positively charged vanadium ions are repulsed by the membrane, which is known as the Donnan exclusion effect, resulting in extremely low vanadium ions cross-over [20]. Low vanadium permeation is the reason for studying AEMs for VRFBs. For example, quaternized Diels–Alder poly(phenylene) membranes showed slower capacity fade than Nafion^®^ [21]. Similarly, a quaternized ammonium functionalized poly(fluorenyl ether) AEM exhibited very low vanadium permeation and achieved 100% coulombic efficiency [22]. In another study, quaternized poly (phthalazinone ether ketone ketone) membranes were prepared and investigated for VRFB suitability showing almost 100% coulombic efficiency at a charging–discharging test for 80 cycles measured at a current density of 80 mA/cm^2^ [23]. Recently, polysulfone-based cross-linked AEMs were applied in VRFBs also showing coulombic efficiencies of 100% after 100 cycles at 50 mA/cm^2^ indicating excellent blocking of vanadium ions [24]. Ren et al. [25] investigated blend membranes of quaternized polysulfone/polyvinylidene fluoride (PVDF) showing improved mechanical properties and performance in VRFBs due to blending. Various studies have investigated the use of amphoteric membranes via acid-base blending for VRFBs [26,27]. Again these blend membranes showed high coulombic efficiencies where the capacity retention for long-time cycling from one of the ImPSf/SPEEK blend membranes was 69% at the 50th cycle measured at a current density of 80 mA/cm^2^ while blend membranes prepared from sulfonated poly(ether ether ketone) and quaternized poly(ether imide) reached 64% at the 100th cycle measured at a current density of 50 mA/cm^2^.

As mentioned previously, blend membranes according to the literature showed improved VRFB performance compared to homopolymer membranes. Previously, results obtained from 2-component blend membranes were published indicating improved performance as aforementioned. In this study, 3-component polymer containing anion-exchange blend membranes (AEBMs) were prepared and characterized in terms of the physical properties, chemical stability, and performance in VRFBs. For the 3-component blend membranes, bromomethylated poly(2,6-dimethyl-1,4-phenylene oxide) (Br-PPO) was used as an anion-exchange precursor with 1,2,4,5-tetramethylimidazole (TMIm), and poly[(1-(4,4′-diphenylether)-5-oxybenzimidazole)-benzimidazole] (PBI-OO) being used as a matrix polymer to improve the mechanical stability. A small amount of a sulfonated polymer (9 to 15% in blend membranes) was added as a third polymer component for ionical cross-linking. The 3-component anion-exchange blend membranes showed significantly improved VRFB performance regarding vanadium ion cross-over when compared to 2-component blend membranes containing only PPO-Br and PBI-OO. One of the 3-component anion-exchange blend membranes did not show any changes in coulombic efficiency during 300 cycles maintaining almost 100%. At the 300th cycle performed at the current density of 40 mA/cm^2^, 77% of capacity had remained. The anion-exchange blend membrane showed significantly longer stability during the open circuit voltage (OCV) test due to the lower vanadium ions cross-over when compared to commercial membranes. Accordingly, the anion-exchange blend membranes would be promising candidates for long-term operation in VRFB applications.

## 2. Materials and Methods

### 2.1. Materials

**Figure 1:**
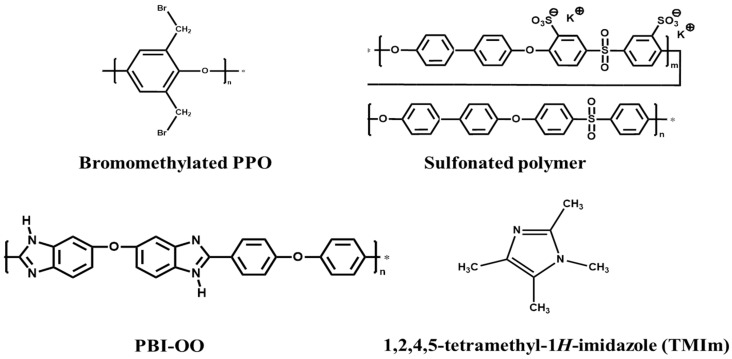
Structures of polymers and TMIm used in this study.

All chemicals were used without further purification. Poly(2,6-dimethyl-1,4-phenylene oxide) (PPO) was purchased from Sigma Aldrich (Munich, Germany), and bromination was conducted in the same way as described previously [28]. Poly[(1-(4,4′-diphenylether)-5-oxybenzimidazole)-benzimidazole] (PBI-OO) was purchased from FuMA-Tech GmbH (Ludwigsburg, Germany). Sulfonated polymer was synthesized as previously reported in the literature [29]. 1,2,4,5-tetramethylimidazole was purchased from TCI chemicals. *N*,*N*-Dimethylacetamide (DMAc) and methanol were obtained from VWR International GmbH (Bruchsal, Germany). Sulfuric acid, potassium hydroxide, 0.1 N standard hydrochloric acid, and sodium hydroxide were purchased from Carl Roth GmbH (Karlsruhe, Germany). Vanadium electrolyte was provided by RIVA GmbH Batteries. The structures used in this study are presented in Figure 1.

### 2.2. Membranes Preparation

**Figure 2:**
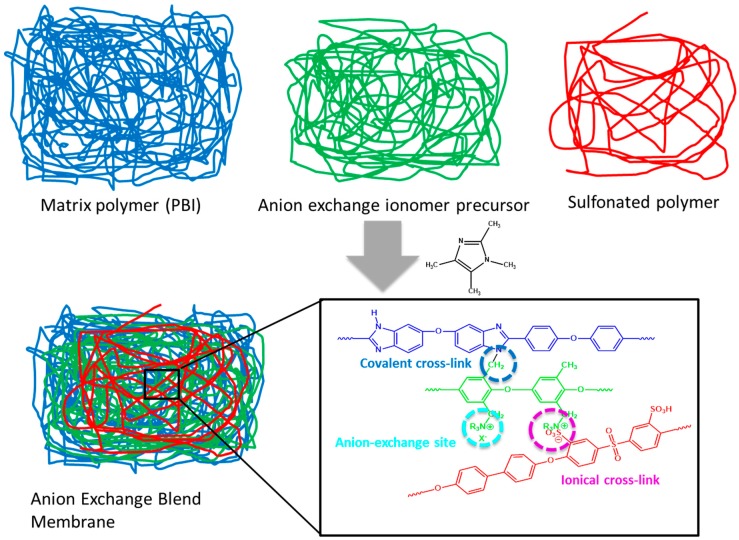
Preparation of anion-exchange blend membranes.

Details of the preparation of the anion-exchange blend membranes are shown in Figure 2. Each polymer solution was prepared separately as a 10 wt % solution in DMAc. Polymer solutions were mixed together, then TMIm was added in to the polymer mixture solution directly in the desired quantities and homogenized. The blend solution was then casted onto a glass plate, followed by solvent evaporation in a convection oven (pre-heated at 40 °C) at 60 °C for 24 h. After drying, the formed membrane was separated from the glass plate by immersion in water. Then membranes were immersed in 1 M sulfuric acid for 1 day at room temperature with one replacement of the sulfuric acid solution during this period. The membranes were repeatedly washed with deionized water at room temperature for 1 day to remove all excess sulfuric acid before being stored in plastic zipper bags before further use.

### 2.3. Membrane Characterization

#### 2.3.1. Ion Exchange Capacity

The membranes were immersed in a 1 M KOH solution for 1 day at 90 °C for ion exchange to hydroxide form. After ion exchange, the membranes were washed with water several times to remove excess KOH. Subsequently, the membranes were immersed in a 60 mL saturated sodium chloride solution to exchange the anion-exchange groups to chloride and stirred for 1 day. Subsequently, 3 mL of a standard 0.1 N hydrochloric acid was added to the saturated sodium chloride solution and stored overnight. The next day, the membranes were washed with 25 mL deionized water and this water was added to the 60 mL saturated sodium chloride solution. The back titration was conducted with a 0.1 N sodium hydroxide solution. Finally, the membrane was repeatedly washed with water and dried at 60 °C. The total IEC was calculated using the following equation.

IEC = (C_HCl_ × V_HCl_ − C_NaOH_ × V_NaOH_)/m_dry_ (1)

where IEC is the ion exchange capacity (OH form, mmol/g), C_HCl_ is the concentration of a hydrochloric acid solution, V_HCl_ is the used volume of a hydrochloric acid solution, C_NaOH_ is the concentration of a sodium hydroxide solution, V_NaOH_ is the added volume of a sodium hydroxide solution, and m_dry_ is the dry weight of the membrane.

#### 2.3.2. Conductivity

A Zahner–elektrik IM6 impedance spectrometer (Kronach, Germany) was used for measuring impedance under ambient atmosphere. The impedance was recorded at room temperature in a 1 M sulfuric acid solution with a frequency range of 200 KHz to 8 MHz in the potentiostatic mode (amplitude 10 mV). The membrane resistance was obtained from the intercept in the real X-axis. The conductivity was determined using the follow equation.

σ = 1/R_sp_ = d/R × A (2)

where σ is the conductivity (mS/cm), R_sp_ is the resistivity (Ω cm), d is the thickness of membrane (cm), R is the ohmic resistance (Ω), and A is the electrode area (cm^2^)

#### 2.3.3. Water Uptake (WU) and Swelling Ratio (SR)

The water uptake and swelling ratio were determined by comparing the membrane weight, length, width, and thickness of a dry and a wetted membrane. A wet membrane sample was cut and the weight, length, width, and thickness was measured after removal of residual water on the surface by blotting with a tissue paper. After drying the membrane at 90 °C for 1 day, the weight, length, width, and thickness were again measured. The water uptake and swelling ratio were calculated using the following equations.

WU (%) = (Wet weight − Dry weight)/Dry weight × 100(3)

SR_L_ (%) = (Wet length − Dry length)/Dry length × 100 (4)

SR_W_ (%) = (Wet width − Dry width)/Dry width × 100 (5)

SR_T_ (%) = (Wet thickness − Dry thickness)/Dry thickness × 100 (6)

#### 2.3.4. Fourier-Transform Infrared Spectroscopy (FT-IR)

FT-IR spectra of the membranes were recorded comprising 64 scans in the wave number range from 4000 to 400 cm^−1^ using a Nicolet iS5 (Thermofisher Scientific, Karlsruhe, Germany) and a diamond attenuated total reflectance (ATR) module.

#### 2.3.5. Gel Content by Extraction

To determine the degree of dissolution of the membrane in an organic solvent, the weight loss was calculated by measuring the weight difference of the membranes before and after DMAc extraction. The dry membrane was kept in DMAc for 4 days at 90 °C and the following 3 days in methanol. The weight loss was determined using the following equation.

Gel (%) = (Weight after/Weight before) × 100(7)

#### 2.3.6. Thermal Stability

To investigate the thermal stability of the membrane, thermal gravimetric analysis (TGA) was done using a dried membrane (dried at 90 °C in convection oven) at a heating rate of 20 °C per minute under O_2_/N_2_ atmosphere (65–70% oxygen) with a NETZSCH TGA, model STA 499C (Selb, Germany).

#### 2.3.7. Chemical Stability

The chemical stability of the membrane was determined using an electrolyte solution (1.6 M VOSO_4_ in 30% H_2_SO_4_). A dry membrane was soaked in this solution at room temperature for 12 days. Subsequently, the membrane was washed repeatedly with water for 1 day before being dried at 90 °C in an oven. The weight loss (WL) of the membrane was determined using the following equation.

WL (%) = [(Weight before − Weight after)/Weight before] × 100(8)

### 2.4. Vanadium Redox Flow Battery (VRFB) Test

**Figure 3:**
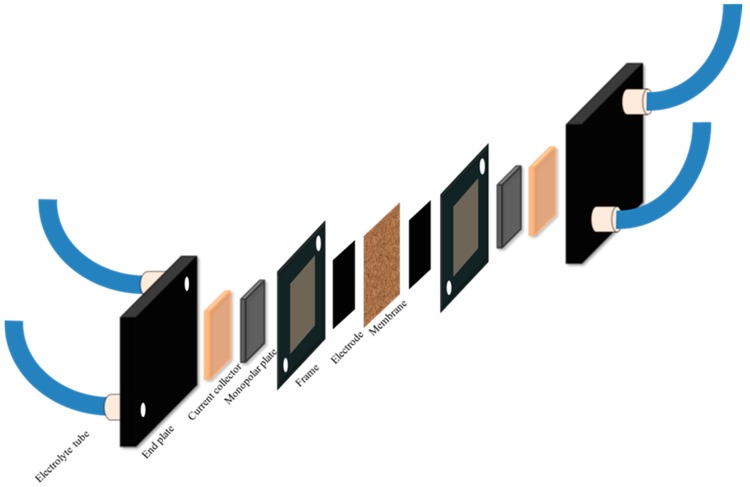
The configuration of vanadium redox flow battery single cell.

A VRFB single cell was manufactured as shown in Figure 3. The anion-exchange blend membrane with an active area of 28 cm^2^ was placed between two carbon felts. Copper plates were used as a current collector. The single cell was assembled between two end plates, and the screws were fastened with a torque of 3.5 Nm. Polyethylene tubes were utilized to supply the electrolytes. Twenty mL of 1.6 M VOSO_4_ in 30% H_2_SO_4_ was filled in tubes of both sides as electrolytes, without pumps and tanks. The cell was first charged to 1.6 V at 40 mA/cm^2^ and discharged to 1.0 V at the same current density, then charged and discharged at different current densities. The open circuit voltage (OCV) was recorded after charging the cell to 1.6 V. A long-term charging–discharging cycles test was conducted with a current density of 40 mA/cm^2^. The coulombic efficiency (CE), voltage efficiency (VE), and energy efficiency (EE) for the process were calculated as follows.

CE (%) = t_d/_t_c_ × 100 (9)

VE (%) = V_d/_V_c_ × 100 (10)

EE (%) = CE × VE × 100(11)

where t_d_ is the discharging time, t_c_ is the charging time, V_d_ is the average discharging voltage, and V_c_ is the average charging voltage.

## 3. Results and Discussion

### 3.1. Preparation and Properties of Anion-Exchange Blend Membranes

Anion-exchange blend membranes composed of two or three polymeric components which had been quaternized with TMIm were prepared. Details of the membranes and their compositions are listed in Table 1. PBI-OO was used as a matrix polymer since polybenzimidazoles are known to possess high mechanical and thermal stabilities [30]. The mechanical stability was further enhanced in the blend system by the covalent cross-linking with Br-PPO [31,32], as shown in Figure 2. Br-PPO was used as an anion-exchange precursor, and the anion-exchange polymer was formed by quaternization of the bromomethyl groups of Br-PPO with TMIm. A sulfonated polymer was used as an ionical cross-linker (sulfonate anions with tetramethylimidazolium cations).

Initially the anion-exchange blend membranes were made using an excess of TMIm (BM-TMIm 1), but the membranes showed turbidity after solvent evaporation. Moreover, they were mechanically unstable when wet due to high water uptake (Figure S1). Thus, the amount of TMIm was reduced to the same equivalent of Br-PPO (except for BM-TMIm 1—see Table 1) resulting in transparent membranes after solvent evaporation and mechanical stability of the water-swollen membranes. Anion-exchange blend membranes were prepared with different ratios between the PBI-OO and Br-PPO. It was subsequently observed that BM-TMIm 2 (Br-PPO:PBI-OO = 7:3 by wt %) was a turbid membrane when dry and mechanically not very stable. Transparent and mechanically stable blend membranes were obtained by reducing the anion-exchange polymer (i.e., Br-PPO amounts). It turned out that blend membranes containing high amounts (more than 52%) of TMIm-quaternized PPO yielded turbid membranes after solvent evaporation, being mechanically instable when wet. It should be noted that the blend membrane composed of Br-PPO and PBI-OO (6:4 by weight percent) without TMIm showed very low conductivity (0.41 mS/cm in 1 M sulfuric acid) and no performance in the VRFBs test, thus, blend membranes in this study, mostly from quaternized groups originated from the quaternization reaction of Br-PPO with TMIm.

**Figure 4:**
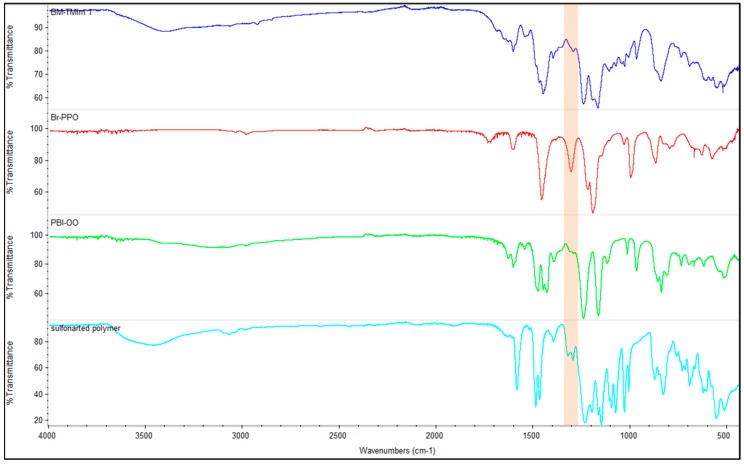
Fourier-Transform Infrared (FT-IR) spectrum of blend-forming polymers and of one anion-exchange blend membrane as an example (BM-TMIm 1).

A structural analyses of the three individual polymers (Br-PPO, S-polymer, PBI-OO) used as well as on one membrane (BM-TMIm 1) were performed using FT-IR (ATR mode) as presented in Figure 4. According to the literature, the peaks of the imidazolium-quaternized PPO were found at 1573, 752, and 3400 cm^−1^ [33]. These peaks are found in blend membranes (Figure S2). However, in the blend membranes these peaks overlap with other polymer peaks and were hence not clearly recognizable. The stretching vibration peaks of the CH_2_Cl group in poly(vinylbenzyl chloride) were found at 675, 709, and 1267 cm^−1^ [34]. Thus, the strong peak at 1302 cm^−1^ in Br-PPO in Figure 4 can be assigned as a stretching vibration of the CH_2_Br group, which is not found in the blend membranes, indicating that no bromomethyl groups were left in the blend membranes after quaternization.

A summary of the properties of the membranes is listed in Table 2. All synthesized anion-exchange blend membranes in this study showed comparable IECs with other published results [35]. It is known that the conductivity of anion-exchange membranes is much lower than proton conductivity of cation-exchange membranes in aqueous solution [22]. Both BM-TMIm 1 and BM-TMIm 2 showed higher ionic conductivities (149 and 144 mS/cm, respectively) than Nafion^®^ (98.5 mS/cm) in acidic media (1 M sulfuric acid), which can be ascribed to the high uptake of sulfuric acid contributing to ionic conductivity. This confirms that conductivities can be achieved that are comparable to cation-exchange membranes. Therefore, the disadvantage of the low conductivity of AEMs can be overcome by adjusting the IECs and electrolyte uptakes when applied in VRFBs, since contribution of conductivity in AEMs is sulfate anions and protons. But in the PEMs, some of the anionic sites are occupied by low mobility vanadium anions [36]. With increasing Br-PPO content in the blend membranes, the conductivity increased while the dimensional stability decreased due to the increased water uptake. The addition of the sulfonated polymer resulted in decreased conductivities but enhanced dimensional stability and higher gel content (see data of BM-TMIm 1-1 and BM-TMIm 6 in Table 2). Thus swelling properties of the anion-exchange blend membranes can be controlled by varying the anion-exchange polymer percent and/or addition of a sulfonated polymer, where a tradeoff has to be sought between the swelling degree and the ionic conductivity.

**Figure 5:**
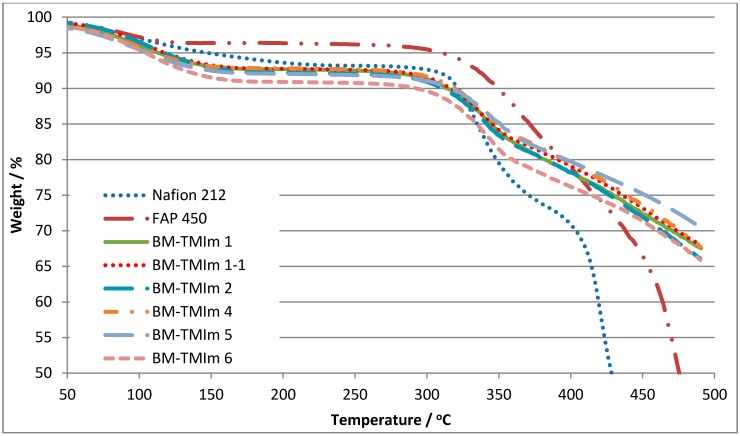
Thermal gravimetric analysis (TGA) traces of membranes in comparison to FAP 450 (anion-exchange membrane produced by Fuma-Tech) and to Nafion^®^ 212.

The thermal stability of the blend membranes as determined using thermal gravimetric analysis (TGA) is shown in Figure 5. The first weight loss step (50–150 °C) is attributed to the water evaporation absorbed in the membranes. The second step starts at 300 °C, which is due to the loss of quaternized imidazolium groups and splitting-off of the sulfonate groups of the sulfonated blend component [37]. Thus, thermal stabilities of 2-component (BM-TMIm 6, 303 °C, loss of quaternized imidazolium) and 3-component (BM-TMIm 1-1, 314 °C, loss of quaternized imidazolium and sulfonated group) blend membranes showed similar thermal stabilities. It is clear from the TGA traces that all the anion-exchange blend membranes had excellent thermal stabilities.

**Figure 6:**
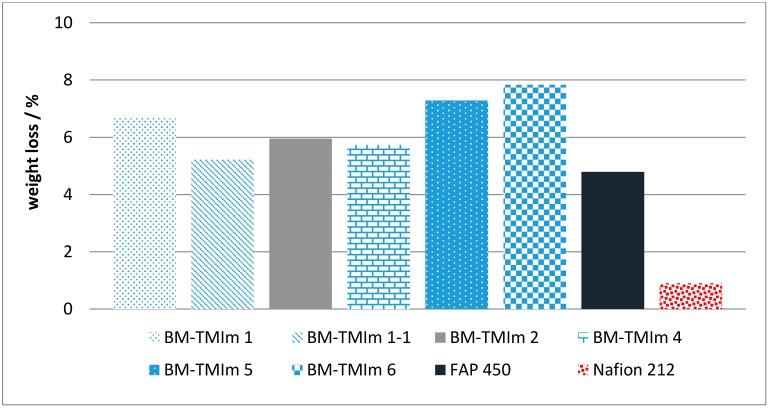
Weight losses after soaking in electrolyte solution at room temperature for 12 days.

By comparing the weight variation of membranes after immersion in electrolyte solution, the relative chemical stability can be evaluated [38]. According to the literature, the proposed degradation pathway for membranes is based on aromatic backbone degradation caused by vanadium peroxo radicals resulting in chain-scission and weight loss by leaching out the degraded polymer chains of the blend components [13]. For the chemical stability test, the membranes were immersed in an electrolyte solution (1.6 M VOSO_4_ in 30% H_2_SO_4_) at room temperature for 12 days. The weight losses before and after the test are shown in Figure 6. All the blend membranes showed weight losses in the range of 5% to 8% which is higher than the weight loss of Nafion^®^ which had an outstanding chemical stability due to its perfluorinated alkyl backbone. The weight loss of the commercial anion-exchange membrane FAP 450 lies in the same range as these of the AEBMs. It can be seen that the 3-component blend membranes (e.g., BM-TMIm 1-1, 5.2%) showed less weight loss than the 2-component blend membrane (BM-TMIm 6, 7.8%). These two membranes are comparable as they were prepared with the same amount of Br-PPO and PBI-OO, with only the sulfonated polymer added in BM-TMIm 1-1 confirming that the additional sulfonated polymer enhanced the chemical stability and mechanical stability.

### 3.2. Vanadium Redox Flow Battery Performance

**Figure 7:**
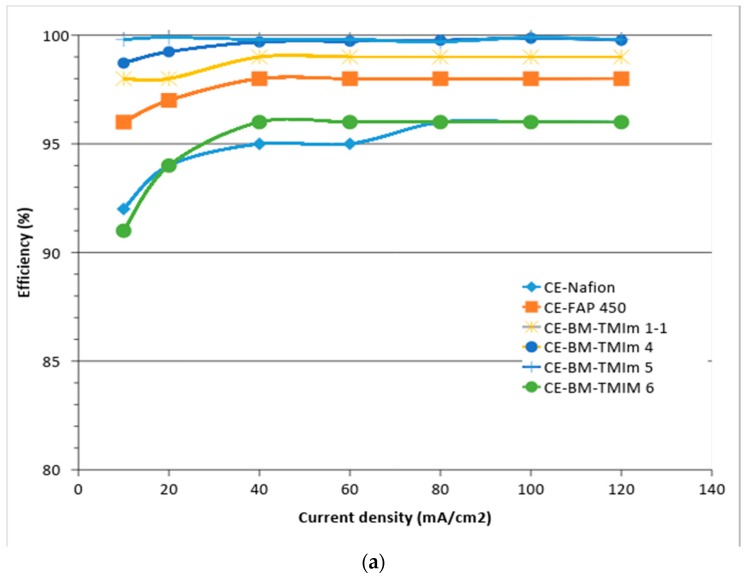

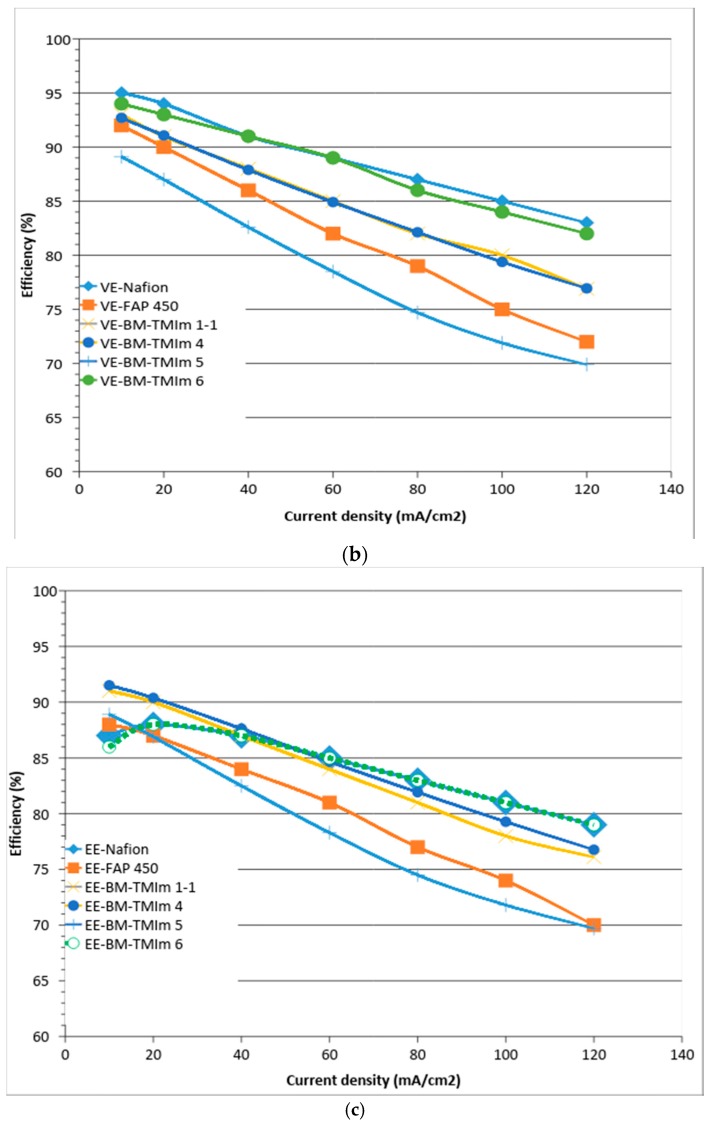
Coulombic efficiency (CE) (**a**); voltage efficiency (VE) (**b**); and energy efficiency (EE) (**c**) of different membranes in VRFBs at various current densities.

The upper limit charge voltage of the single cell was set to 1.6 V to avoid corrosion of the electrodes [39]. The performance of the different membranes was recorded using a single VRFB cell. Due to mechanical membrane failure during single cell preparation (broken, BM-TMIm 1) and a very fast cross-over of electrolyte (BM-TMIm 2) in the VRFB caused by high water uptake and therefore low dimensional stabilities of blend membranes, the performance of BM-TMIm 1 and BM-TMIm 2 were not further measured in battery tests. Figure 7a–c shows the respective CE, VE, and EE of the VRFB as a function of the current density. In general, the CE of VRFBs is influenced by vanadium ions permeation, possible side reactions, and electrode corrosion [40]. Since the VRFB setup was identical for all single cell performance measurements in this study, the side reactions and electrode corrosion should be similar for all battery measurements. Therefore, the main contribution to the CE is the vanadium ions cross-over.

All anion-exchange blend membranes have shown higher CEs than Nafion^®^ due to the Donnan exclusion effect mentioned previously. The 3-component AEBMs had a higher CE than the 2-component AEBMs in VRFBs indicating less vanadium ions cross-over via enhanced dimensional stability. These membranes also showed an increased CE as a function of current density due to the decreased time allowed for vanadium permeation. Hence the 3-component AEBMs had improved vanadium ions blocking properties than the 2-component AEBMs. The VE of all membranes decreased with increasing current density due to the increasing over voltage at the electrodes [41]. The VE is affected by the ohmic resistance which corresponds to the ionic conductivity [40]. Nafion^®^, as expected, showed the highest VE of all the tested membranes in VRFBs due to its high cation conductivity and BM-TMIm 5 showed lowest VE due to the lowest conductivity among the tested membranes. However, it can be seen that the differences of the VEs between BM-TMIm 1-1 or BM-TMIm 4 and Nafion^®^ were small despite the higher conductivity of Nafion^®^ 212, compared to that of most of the blend membranes. Moreover, VEs of BM-TMIm 1-1 and BM-TMIm 4 showed almost the same value despite the higher conductivity of BM-TMIm 1-1, compared to that of BM-TMIm 4. These results suggest that the ionic conductivity in VRFBs is not a critical parameter. The overall energy efficiencies (EE) of AEBMs showed higher values than that of FAP 450 and comparable values to Nafion^®^ irrespective of the current densities. The EE of BM-TMIm 4 was slightly higher than that of Nafion^®^ below a current density of 50 mA/cm^2^, which allows the conclusion that this membrane should be operated in a VRFB at moderate current densities.

**Figure 8:**
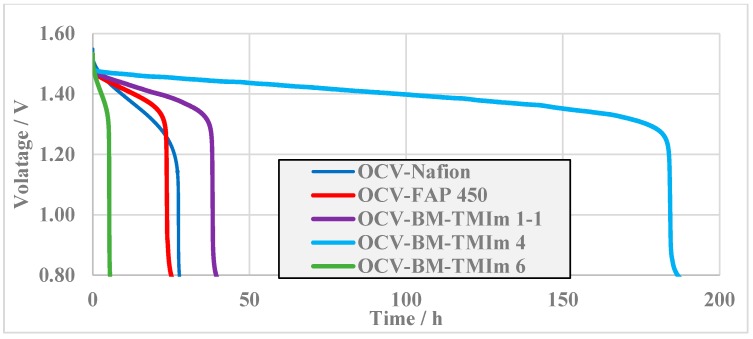
The self-discharge of the VRFBs with different membranes.

Open circuit voltage measurements of VRFBs are often used as an indirect method to investigate the vanadium ions cross-over [27]. Hence, a self-discharge test was run by measuring the OCV as a function of time (Figure 8). It is clear that complete cross-over of vanadium ions through the membranes cannot be avoided because of the diffusion of vanadium ions through the membranes caused by different concentrations of vanadium ion oxidation states between the two electrolytes [42]. In all cases, a sudden OCV drop was observed which can be explained by the disappearance of VO_2_^+^ in the positive electrolyte originating from vanadium ions cross-over. Firstly, the single cell was charged to 1.6 V with a current density of 40 mA/cm^2^ followed immediately by the self-discharge test. While most membranes, including the commercial membranes, showed a sudden drop before 40 h, BM-TMIm 4 had a drop off only after 184.3 h correlating with results of coulombic efficiency originating from the reduced vanadium ions cross-over. It seems that self-discharge is influenced by water uptakes. Higher water uptakes mean higher electrolyte uptake resulting in faster vanadium ions diffusion through the electrolyte. The water uptakes of blend membranes are found to be in the order of BM-TMIm 6 (58%) > BM-TMIm 1-1 (47%) > BM-TMIm 4 (31%) and self-discharge times were 5.3, 38.3, 184.3 h, respectively, as shown in Figure 8. Thus, it can be concluded that the AEMs should possess proper water uptakes for VRFB application (it should be noted that a low water uptake leads to a low conductivity).

**Figure 9:**
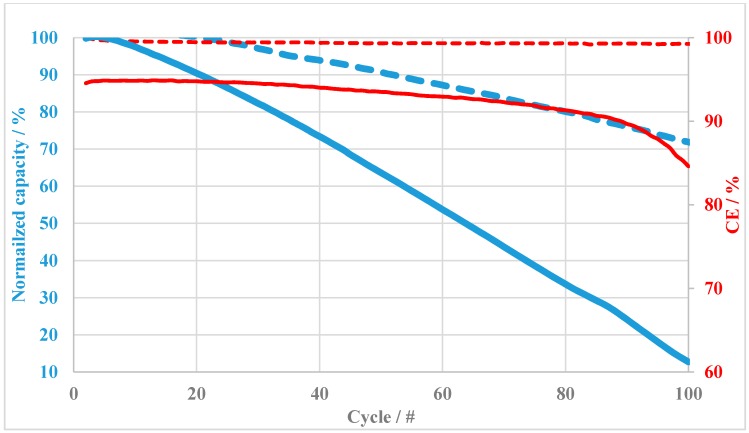
Charging–discharging cycles tests of blend membranes BM-TMIm 1-1 and BM-TMIm 6.

To compare the VRFB performance, charging–discharging cycles tests of the two anion-exchange blend membranes BM-TMIm 1-1 and BM-TMIm 6 were conducted (Figure 9). These two were selected to compare the performances of VRFB between 2-component and 3-component blend membranes. The 2-component blend membrane (BM-TMIm 6) almost completely lost its capacity (13% remaining at 100th cycle), while the 3-component blend membrane BM-TMIm 1-1 had a residual capacity of 72% after the same number of cycles. This test is correlating with the self-discharge results depicted in Figure 8. It can be concluded that 3-component blend membranes displayed a significantly reduced vanadium ions cross-over in the VRFB when compared to the 2-component blend membrane. It is clear that the ionical cross-linking via the sulfonated polymer not only enhanced the blocking of vanadium ions but also increased the dimensional stability.

**Figure 10:**
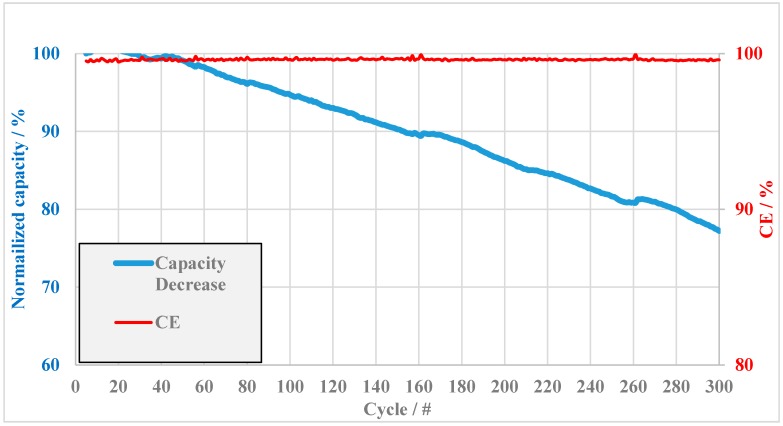
Long-term charging–discharging cycles test with BM-TMIm 4 membrane.

Because BM-TMIm 4 possessed the longest self-discharge time of all the tested membranes in this study, a long-term charging–discharging cycles test was conducted with this blend membrane (Figure 10). It turned out that the coulombic efficiency remained almost 100% (99.6–99.5%) over 300 cycles. According to Figure 9 and Figure 10, the BM-TMIm 4 membrane showed less capacity decay than BM-TMIm 1-1. This result suggests that by adjusting anion-exchange polymer proportion in the blend membrane, improved performance in VRFBs can be achieved. According to Figure 10, the capacity retention of BM-TMIm 4 measured at a current density of 40 mA/cm^2^ was 95%, 86%, and 77% at the 100th, 200th, and 300th cycle, respectively. Compared with values from other VRFB studies of membranes in the literature, it is obvious that the anion-exchange blend membranes in this study showed improved capacity retention, e.g., when using the imidazole-based amphoteric membrane ImPSf/SPEEK 17% the capacity retention was 69% at the 50th cycle with a current density of 80 mA/cm^2^ [26], and the capacity retention of an acid-base blend membrane of S/Q-15 was 64% at the 100th cycle at a current density of 50 mA/cm^2^ [27]. The outstanding capacity retention of the anion-exchange blend membrane presented in this study suggests that this membrane type is a promising candidate for vanadium redox flow battery application if the proportion of the components in the blend membrane and the cross-linking density (covalent and/or ionical) is carefully adjusted.

## 4. Conclusions

A series of anion-exchange blend membranes (AEBMs) having similar thicknesses (wet in 1 M H_2_SO_4_, applied in VRFBs) were fabricated and characterized ex-situ and in-situ in a vanadium redox-flow battery setup. The 3-component AEBMs were composed of bromomethylated PPO, PBI-OO, and a sulfonated polyethersulfone polymer, where the bromomethyl groups of the PPO blend component were quaternized with TMIm. The third component in the blend membranes, the sulfonated polymer, enhanced the chemical stability and dimensional stability of the blend membranes and reduced the vanadium ions cross-over, resulting in improved redox flow battery performance. One of the prepared 3-component blend membranes (BM-TMIm 4) showed an excellent coulombic efficiency of almost 100% after 300 charging–discharging cycles with a significant capacity retention of 77% of the initial value after 300 cycles at a current density of 40 mA/cm^2^. Therefore, it can be concluded that the investigated 3-component AEBMs are promising candidates for long-term operation as an energy storage system in VRFBs if the proportion and type of the different components in the blend is carefully adjusted. In ongoing research on VRFB membranes, BM-TMIm 5 is showing very long self-discharge time as elucidated in our lab and will be reporting soon along with further types of anion-exchange blend membranes where the type and/or proportion of the different components in the anion-exchange blend membranes, including the type of the halomethylated precursor, the type of the tertiary amine for quaternization, the type of the matrix polymer, and the type of the sulfonated or phosphonated ionically cross-linking polymer, will be varied.

## Figures and Tables

**Table 1:** The compositions of the anion-exchange blend membranes.

Entry	Br-PPO **/wt %	PBI-OO ***/wt %	S-Polymer ****/wt %	TMIm ***** (Equivalent)
BM-TMIm * 1	52	35	13	3
BM-TMIm 1-1	52	35	13	1
BM-TMIm 2	60	26	15	1
BM-TMIm 4	45	45	11	1
BM-TMIm 5	36	55	9	1
BM-TMIm 6	60	40	0	1

* Abbreviations: BM-TMIm: blend membranes with 1,2,4,5-tetramethylimidazole; ** Br-PPO: Bromomethylated poly(2,6-dimethyl-1,4-phenylene oxide); *** PBI-OO: poly[(1-(4,4′-diphenylether)-5-oxybenzimidazole)-benzimidazole]; **** S-Polymer: sulfonated polymer; ***** TMIm: 1,2,4,5-tetramethylimidazole. For structures of polymers, see Figure 1.

**Table 2:** The properties of anion-exchange blend membranes.

Entry	IECs (OH Form)	Conductivities (mS/cm)	Gel (%)	Dimensional Stability				T _onset_ (°C)	Thickness (µm, Wet in 1 M H_2_SO_4_)
				WU (%)	SR_L_ (%)	SR_W_ (%)	SR_T_ (%)		
BM-TMIm 1	2.71	149	95	71	31	29	16	281	89
BM-TMIm 1-1	3.26	40.9	95	47	19	18	15	314	71
BM-TMIm 2	3.04	144	94	105	n. a. **	n. a. **	n. a. **	320	92
BM-TMIm 4	3.41	21.0	94	31	12	12	9	306	57
BM-TMIm 5	2.93	13.7	92	33	12	11	8	306	66
BM-TMIm 6	3.39	65.2	92	58	21	20	15	303	63
FAP 450 *	2.18	35.2	- ***	19	9	8	9	304	58
Nafion^®^ 212	0.88 (H form)	98.5	-	8	7	9	3	299	53

* FAP 450: anion-exchange membrane produced by Fuma-Tech; ** not applicable due to mechanical failure; *** extremely swelling in DMAc.

## Supplementary Materials

The following are available online at http://www.mdpi.com/2077-0375/8/2/33/s1, Figure S1: Anion exchange blend membranes tested in this study, Figure S2: Comparison of FT-IR spectrum of AEBMs with Br-PPO.
