# Supplementary material for: Application of Novel Anion-Exchange Blend Membranes (AEBMs) to Vanadium Redox Flow Batteries
Source: Membranes (Basel). 2018 Jun 19;8(2):33. doi: 10.3390/membranes8020033 (PMC6027430; doi:10.3390/membranes8020033)
Supplement: Supplementary file 1 [file membranes-08-00033-s001.pdf]

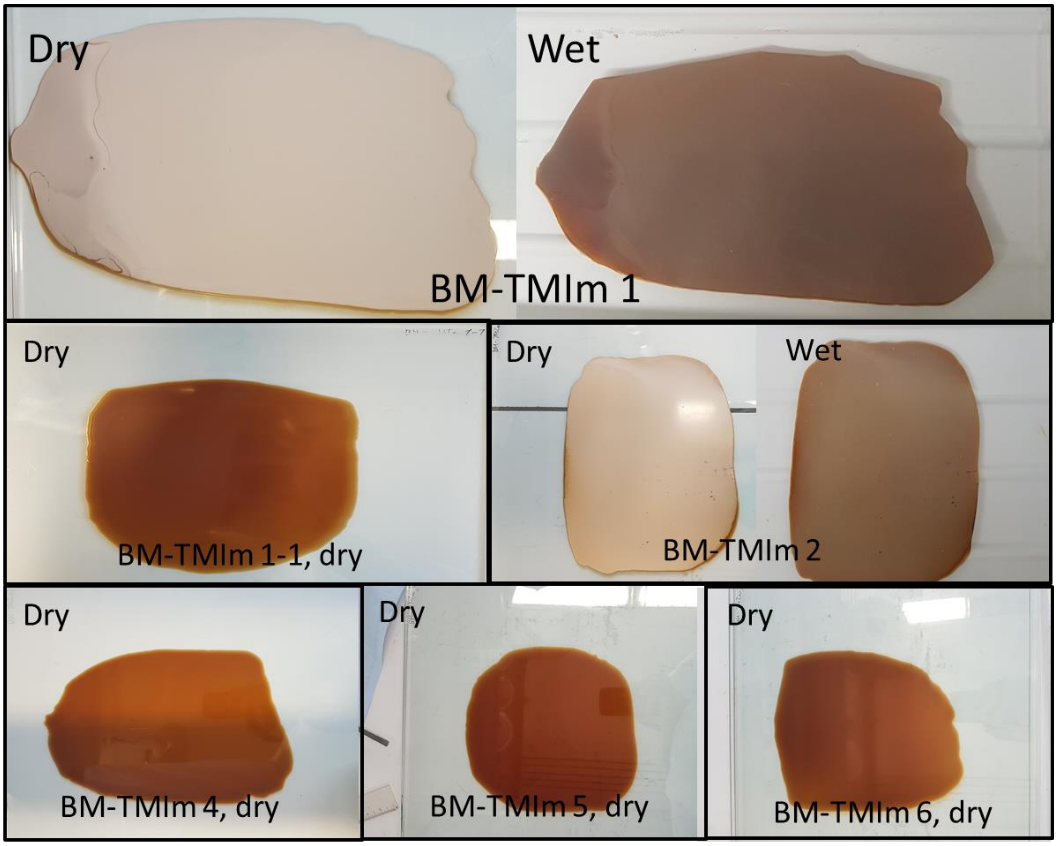

Figure S1. Anion exchange blend membranes tested in this study.

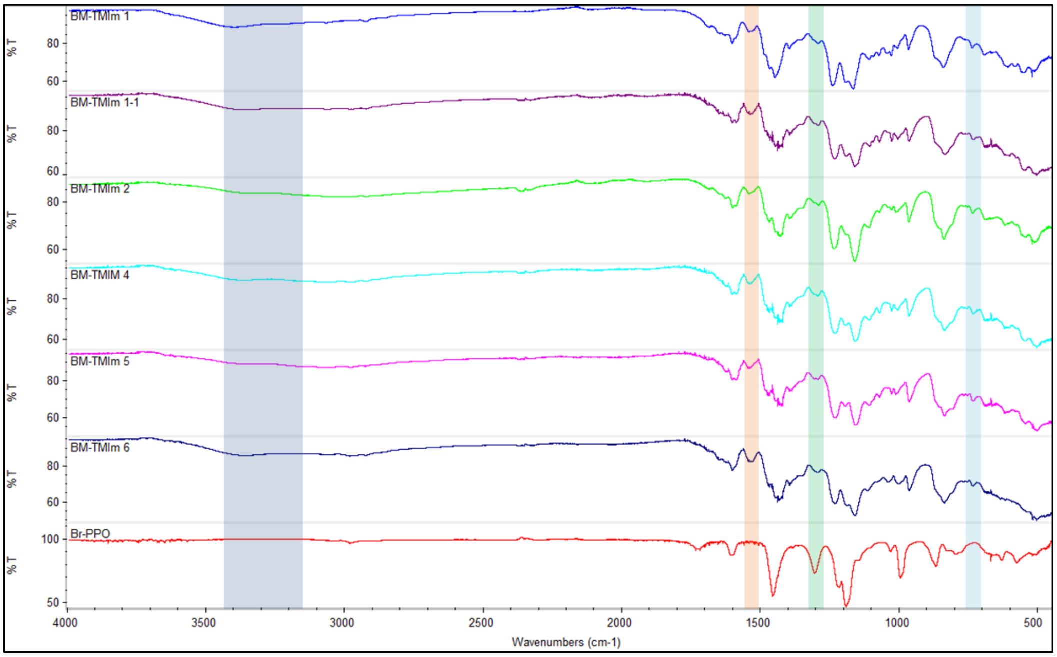

Figure S2. Comparison of FT-IR spectrum of AEBMs with Br-PPO.
